# Lodging Resistance of Japonica Rice (*Oryza Sativa* L.): Morphological and Anatomical Traits due to top-Dressing Nitrogen Application Rates

**DOI:** 10.1186/s12284-016-0103-8

**Published:** 2016-07-01

**Authors:** Wujun Zhang, Longmei Wu, Xiaoran Wu, Yanfeng Ding, Ganghua Li, Jingyong Li, Fei Weng, Zhenghui Liu, She Tang, Chengqiang Ding, Shaohua Wang

**Affiliations:** Jiangsu Collaborative Innovation Center for Modern Crop Production/National Engineering and Technology Center for Information Agriculture/ Key Laboratory of Crop Physiology and Ecology in Southern China, Nanjing Agricultural University, Nanjing, 210095 China; Chongqing Academy of Agricultural Sciences/Chongqing Ratooning Rice Research Center, Chongqing, 402160 China

**Keywords:** Japonica rice, Lodging resistance, Morphological and anatomical traits, Stem strength, Top-dressing N application

## Abstract

**Background:**

Lodging in rice production often limits grain yield and quality by breaking or bending stems. Excessive nitrogen (N) fertilizer rates are the cause of poor lodging resistance in rice, but little is known about the effect of top-dressing N application rates on the mechanical strength of japonica rice plants, especially how the anatomical structure in culms is affected by N. In this study, field experiments on two japonica rice varieties with three top-dressing N application rates, 0 kg N ha^−1^ (LN), 135 kg N ha^−1^ (MN), and 270 kg N ha^−1^ (HN) as urea, were conducted. Wuyunjing23, a lodging-resistant japonica rice cultivar and W3668, a lodging-susceptible japonica rice cultivar were used. The lodging index, breaking strength, morphological and anatomical traits in culms were measured in this study.

**Results:**

The visual lodging rate in japonica rice differed remarkably between genotypes and top-dressing N treatments. The higher lodging index of rice plants was primarily attributed to the weak breaking strength of the lower internodes. The longer elongated basal internodes were responsible for higher plant height and a higher lodging index. Correlation analysis showed that breaking strength was significantly and positively correlated with the thickness of the mechanical tissue but was significantly and negatively correlated with the inner diameter of the major axis (b_2_). With increasing top-dressing N rates, the sclerenchyma cells of the mechanical tissues and the vascular bundles of the Wuyunjing23 cultivar varied little. The plant height, inner diameter of the minor axis (a_2_) and b_2_ increased significantly, but the area of the large vascular bundle (ALVB) and the area of the small vascular bundle (ASVB) decreased significantly and resulted in lower stem strength and a higher lodging index under higher top-dressing N conditions. The culm diameter of the W3668 cultivar increased slightly with no significant difference, and the sclerenchyma cells in the mechanical tissues and vascular bundles showed deficient lignifications under high top-dressing N conditions. Moreover, the ALVB and the ASVB decreased significantly, while the area of air chambers (AAC) increased rapidly.

**Conclusions:**

An improvement in the lodging resistance of japonica rice plants could be achieved by reducing the length of the lower internodes, decreasing the inner culm diameter and developing a thicker mechanical tissue. Top-dressing N application increased the plant height and inner culm diameter and decreased the ALVB and the ASVB of the Wuyunjing23 cultivar and caused deficient lignified sclerenchyma cells, lowered the ALVB and the ASVB, and increased the AAC of the W3668 cultivar resulting in weaker stem strength and a higher lodging index.

## Background

Lodging refers to the permanent displacement of stems from an upright position. The proportionality between the physical strength of lower internodes and the weight of the upper parts determines a plant's vulnerability to lodging (Mulder 1954). In cereals, the reduction of plant height has been the main target for improving lodging resistance. Plant breeders have reduced lodging risks by introducing semi-dwarf traits to produce shorter cultivars, known as the Green Revolution (Khush, 1999). Recent studies, however, indicate that the plant height of semi-dwarf rice and wheat may limit canopy photosynthesis and biomass, thereby limiting grain yield (Kuroda et al., 1989; Gent, 1995). Another problem with semi-dwarfism is that the gene regulating the semi-dwarf trait may exhibit negative pleiotropic effects on stem morphology. For example, semi-dwarf 1 (*sd1*) gibberellin (GA) synthesis is used for the reduction of rice plant height but also decreases stem stiffness by lowering the culm diameter and thickness (Okuno et al., 2014). Ma et al. (2004) studied the optimum length of internodes in rice that would increase lodging resistance and found that plant height is not a primary factor for lodging risks (Islam et al., 2007).

According to a previous study by Sterling et al. (2003), the lodging of cereal plants can be classified into two types. Root lodging results from intact and unbroken culms leaning from the crown due to a failure of root anchorage in the ground, while stem lodging refers to the bending or breaking of the lower culm internodes as a result of excessive bending pressure at the higher internodes. Stem strength i.e., the bending or breaking strength of the culm, is important for stem lodging resistance, particularly for the basal internodes of crops (Zuber et al., 1999). Thus, the new major focus for improving lodging resistance and grain yield is increasing the stem strength of the lower internodes of rice plants (Yao et al., 2011; Zhang et al., 2013; Chen and Du 2010).

The stem strength of cereal plants is primarily determined by plant architecture (morphological traits and anatomical structure). In particular, anatomical structure is a consequence of plant growth and development at the cellular level, such as cell division, cell growth and cell spatial arrangement, and is closely related to environmental factors (Huber et al., 2013). In maize, a shading condition induces a reduction in the thickness of the mechanical tissue, the number of vascular bundles and the area of the xylem and phloem, which can be attributed to the weak mechanical strength of the stem (Cui et al., 2012). The response in plant architecture of wheat to high planting density is characterized by a longer and slender stem with low-density tissue, resulting in poor lodging resistance (Zheng et al., 2013). The rice and wheat plant that have a high resistance to stem breaking and lodging have a higher outer diameter, culm wall thickness, thickness of mechanical tissue and a larger number of large and small vascular bundles in rice and wheat (Kong et al., 2013; Duan et al., 2004; Fu et al. 2013). However, Dunn and Briggs (1989) and Kelbert et al. (2004) argue that among barley and wheat cultivars, the thickness of sclerenchyma tissue is not associated with a difference in lodging resistance.

The application of nitrogen (N) fertilizer is one of the important measures for the improvement of rice grain yields. However, excessive rates of N application are the cause of lodging. In previous studies, high N application rates resulted in poor lodging resistance in rice and wheat by increasing tiller numbers, the length of lower internodes, gravity centre height and plant height and decreasing the dry weight per cm in culms, the leaf sheath strength and the breaking strength (Yang et al., 2009; Wang et al., 2012; Zhang et al., 2013; Li et al., 2013). Shi et al. (2008) showed that the lodging index decreases then increases with N application rates, which means that the risk of lodging risks in rice plants is smallest at a middle N fertilizer rate. Quang Duy et al. (2004) suggests that a sparse planting density accompanied by small amounts of N fertilizer in the early growth stages effectively increases lodging resistance in rice plants.

Previous researchers have compared the effects of different N application rates or N application rates combined with different transplanting density conditions on characteristics related to lodging resistance. To our knowledge, top-dressing N is an important environmental factor that affects biomass production, grain yield and lodging resistance. However, little is known about top-dressing N fertilizer applications on the mechanical strength of japonica rice basal internodes, especially how the anatomical structure of culms is affected by N. The objective of this paper was to determine the effects of top-dressing N fertilizer on the anatomical structure in culms and its relation to lodging resistance. This provides a theoretical basis for how to enhance the mechanical strength of the stem to prevent japonica rice lodging and improve grain yield and quality.

## Methods

### Experimental Site

Field experiments were carried out at Danyang, Jiangsu Province, China (32°00’N, 119°32’E, 7 m altitude) during the rice growing season from late May to late October in 2013 and 2014. This area is classified as having a subtropical monsoon climate, and the soil at the site is classified as alluvial loamy soil. The soil layer is 0–20 cm deep, has a pH of 6.2, a total N content of 0.973 g kg^−1^, available phosphorus content of 13.60 mg kg^−1^, available potassium content of 93.50 mg kg^−1^, and organic matter content of 17.15 g kg^−1^.

### Experiment Design and Crop Management

Field experiments were arranged in a randomized block design with three replications in 2013 and 2014. Two rice cultivars (*Oryza sativa L.*) were used in this study: Wuyunjing23, a lodging resistant cultivar and W3668 a lodging susceptible cultivar. Seeds were sown in nursery boxes on 26 May. On June 24, when the seedlings were at the fifth-leaf age, three seedlings were transplanted per hill. The planting density was 25 hills m^−2^, at a spacing of 30 cm × 13.3 cm. Three nitrogen fertilizer rates, 135 kg N ha^−1^ (LN), 270 kg N ha^−1^ (MN), and 405 kg N ha^−1^ (HN) as urea, were defined and applied as follows: 67.5 kg N ha^−1^ as urea was applied 1 day before transplanting, and 67.5 kg N ha^−1^ as urea was applied 7 days after transplanting in all plots; then, three top-dressing N fertilizer rates 0 kg N ha^−1^ (LN), 135 kg N ha^−1^ (MN), 270 kg N ha^−1^ (HN) were applied as urea two times (at the panicle initiation stage and when the 2nd leaf from the top was fully extended) by using 60 and 40 % at each stage, respectively. Phosphorus at 90 kg P_2_O_5_ ha^−1^ as single superphosphate was applied 1 day pre-transplantation, and potassium at 120 kg K_2_O ha^−1^ as potassium chloride was applied 1 day pre-transplantation and before panicle initiation using 50 and 50 % at each stage in all plots. Each plot size was 24 m^2^ (4 m × 6 m). Each plot between different fertilizer rates was surrounded by 30 cm-wide ridges, which were covered with plastic film. The plastic film was installed to a depth of 15 cm below the soil surface 2 days before transplanting.

Except for the chemical fertilizer application described above, crop management and experimental methods were similar for both sites and years. The water management was based on alternate wetting and drying irrigation methods (Liu et al., 2013). In brief, plots were kept at a 2–3 cm water level during the 1st week after transplantation, anthesis, and the timing for N top-dressing. At other growth stages, fields were not irrigated until the soil water potential reached between −10 kPa and −15 kPa at a 15–20 cm depth. Herbicide was used to control weeds. Insects and disease were intensively controlled by chemicals to avoid biomass and yield loss.

### Indicators and Measuring Method

The visual lodging rate was measured at a mature grain stage using the formula: visual lodging rate = the lodging area in plot / the plot area × 100 % as reported by Lu et al. (2014). Culm characters related lodging resistance, by using 15 representative main stems from each plot, was measured at 30 days after heading according to the method by Islam et al. (2007). Plant height (the length between the plant base and the panicle tip), the length of the panicle (N_0_), and the first (N_1_), second (N_2_), third (N_3_), fourth (N_4_), and fifth (N_5_) internodes from the top were measured. The lodging index (LI) and breaking strength (M) of N_4_ internodes were determined according to the method of Ookawa (1992) because stem lodging usually occurs at the lower internodes (Kashiwagi et al., 2005). In brief, the breaking force of N_4_ internodes with the leaf sheath was measured with a plant lodging tester (digital force gauge) by using a three point bending method. The distance between the fulcra of the tester was set at 8 cm. The centre of the internode, where the breaking resistance was measured, was aligned horizontally with the middle point between the two fulcra. After measuring the breaking force, the stem was cut at the breaking point of the N_4_ internode, and the stem length (SL), from the broken point to the panicle tip, and the fresh weight (FW), from the broken point to the panicle tip, was measured. In addition, the culm diameter and culm wall thickness were also measured after removing the leaf sheath. The outer and inner diameter of the minor axis in an oval cross-section (a_1_ and a_2_) and the outer and inner diameter of the major axis in an oval cross-section (b_1_ and b_2_) were also measured. The breaking strength and lodging index were calculated using the following formulas: breaking strength (M) = breaking load (kg) ×  L/4 (cm) × 1000, where L is the distance between two support pillars (8 cm), and lodging index (LI) = SL (cm) × FW (g) / Breaking strength × 100, where SL is the stem length from the broken point to the panicle tip and FW is the fresh weight from the broken point to the panicle tip.

To observe the anatomical structure within the stem, the middle portions of the culm tissue in the heading stage were excised with a razor and immediately placed in a fixing solution (70 % ethanol: 5 % acetic acid: 3.7 % formaldehyde) for 24 h. Then, the paraffin sections were processed according to the method by Du et al. (2013). The following anatomical characteristics were measured and analysed using a statistical software package attached to the fluorescence microscope (Axioskop 40 with UV excitation, ZEISS): the number of large vascular bundles (LBVB), the area of large vascular bundles (ALVB), the number of small vascular bundles (NSVB), the area of small vascular bundles (ASVB), the number of air chambers (NAC), the area of air chambers (AAC) and the thickness of mechanical tissue (TMT). To observe any lignifications of culm tissue (Li et al., 2009), fresh hand-cut sections (20 μm thick) were incubated for 10 min in phloroglucinol solution (1 % in ethanol:water [70:30, v / v]; Sigma); then, the phloroglucinol was removed, and the sections were treated with 18 % HCl for 5 min and then photographed under a light microscope (model DM4000B; Leica, Germany).

To test the differences among different treatments, variance analysis was performed using SPSS 20.0 statistical software. The means of the treatments were compared based on the significant difference test (SSR) at a 0.05 probability level. The standard deviation of the means was calculated using Microsoft Excel 2007 software for Windows.

## Results

### Visual Lodging Rate

The visual lodging rate of the Wuyunjing23 cultivar was significantly lower than that of the W3668 cultivar (8.3 % vs 71.6 %, mean value of 2 years) in both years. Furthermore, the response of the two varieties to top-dressing N, in terms of the visual lodging rate, was largely different. For Wuyunjing23, the visual lodging rate happened in 2014 only and increased from 0.0 to 39.2 % with increasing top-dressing N. For W3668, the visual lodging rate varied between 1.3 and 92.5 % in 2013 and 71.9 and 95.8 % in 2014 under different N application rates (data not shown).

### Lodging Index and Breaking Strength

**Fig. 1:**
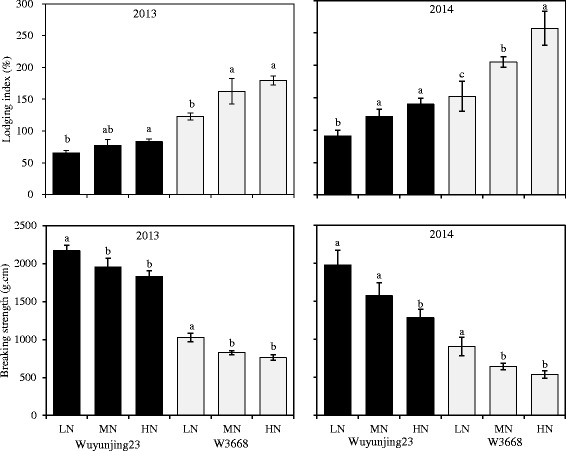
Comparison of the lodging index and breaking strength of the N_4_ internodes 30 days after heading in japonica rice cultivars under different nitrogen rates. Different lowercase letters represent significant differences (*P* < 0.05) relative to the LN treatments for Wuyunjing23 and W3668, respectively

The lodging index of Wuyunjing23 was significantly lower than that of W3668, whereas the breaking strength of Wuyunjing23 was higher (Fig. 1). With an increase in the top-dressing N application rates, the lodging index of the Wuyunjing23 cultivar increased by 27.3 % (2013) and 53.8 % (2014), and that of the W3668 cultivar increased by 46.3 % (2013) and 69.1 % (2014). By contrast, the breaking strength in the Wuyunjing23 cultivar decreased by 15.6 % (2013) and 34.9 % (2014), and that of the W3668 cultivar decreased by 25.9 % (2013) and 40.4 % (2014).

### Culm Morphological Traits

**Fig. 2:**
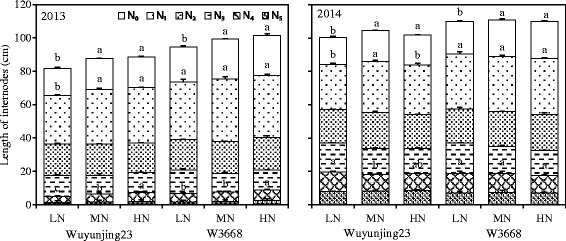
Configuration length of internodes in two japonica rice cultivars under different nitrogen rates (N_0_, panicle length; N_1_, N_2_, N_3_, N_4_ and N_5_ denote the first to the fifth internode from the top, respectively.) Different lowercase letters represent significant differences (*P* < 0.05) relative to the LN treatments for Wuyunjing23 and W3668, respectively

Figure 2 shows the length of the lower internodes (N_4_ + N_5_), upper internodes (N_1_ + N_2_ + N_3_) and panicles (N_0_). Compared with W3668, the lengths of the lower internodes, upper internodes and panicles in Wuyunjing23 were significantly lower by 8.7, 7.5 and 20.5 %, respectively. In 2013, as the top-dressing N application rate increased the length of each elongated internode, and thus, the plant height increased. For instance, the length of the lower internodes, upper internodes and panicles increased by 14.9, 4.0, 46.0 % in Wuyunjing23 and 13.2, 3.0, 29.8 % in W3668. However, no significant difference in the length of each elongated internode was obtained in 2014 under different N treatments. Compared with 2013, the lower internodes in 2014 increased by 12.5 cm in the Wuyunjing23 cultivar and 10.7 cm in the W3668 cultivar, which resulted in an increase in total culm length and plant height.

Compared with W3668, the plant height of Wuyunjing23 was lower by 12.8 and 7.1 % in 2013 and 2014, respectively. By contrast, culm diameter and culm wall thickness of the Wuyunjing23 cultivar were significantly higher (Table 1). The culm wall thickness was almost identical under different N treatments in both years. With higher top-dressing N application rates, the culm diameter and the plant height were significantly increased. In addition, as shown by variance analysis (Table 1), top-dressing N significantly increased a_1_ (* F* = 9.5, *P* < 0.01), a_2_ (*F* = 18.7, *P* < 0.01) and b_2_ (*F* = 24.4, *P* < 0.01). Additionally, the interaction between the cultivar and N had significant effects on a_2_ (*F *= 6.1, *P *< 0.01) and b_2_ (*F *= 5.2, *P* < 0.05). Compared with 2013, the culm diameter in 2014 was significantly lower, which resulted primarily from a reduction of a_1_ (*F *= 45.3, *P* < 0.01) and b_1_ (*F* = 39.4, *P* < 0.01), but plant height increased.

**Table 1:** Culm morphology traits of the N_4_ internode culm and plant height in two japonica rice cultivars under different nitrogen rates

Treatments	CD (mm)	CWT (mm)	b_1_ (mm)	a_1_ (mm)	b_2_ (mm)	a_2_ (mm)	PH (cm)	GCH (cm)
2013								
Wuyunjing23								
LN	4.96a	0.76a	5.26a	4.68b	3.63b	3.25b	81.6b	39.5a
MN	5.01a	0.82a	5.30a	4.78a	4.04a	3.46a	87.5a	39.7a
HN	4.96a	0.73a	5.22a	4.71ab	4.00a	3.50a	88.6a	39.8a
W3668								
LN	4.24a	0.64a	4.50a	4.00b	3.56a	2.96b	94.7b	43.9b
MN	4.25a	0.64a	4.50a	4.11a	3.69a	3.06ab	99.2a	45.9a
HN	4.30a	0.65a	4.46a	4.16a	3.73a	3.16a	101.4a	47.2a
2014								
Wuyunjing23								
LN	4.74a	0.77a	5.02a	4.46a	3.59b	2.97b	101.5b	46.8a
MN	4.84a	0.82a	5.13a	4.55a	3.82a	3.42a	105.5a	47.8a
HN	4.93a	0.81a	5.16a	4.60a	3.88a	3.19ab	103.5ab	48.2a
W3668								
LN	4.02a	0.65a	4.14b	3.78b	3.25b	2.80a	110.9a	49.5a
MN	4.03a	0.65a	4.19b	3.87b	3.26b	2.88a	112.2a	50.6a
HN	4.19a	0.64a	4.34a	4.04a	3.39a	2.89a	111.2a	51.3a
Analysis of variance								
Year (Y)	24.5^b^	1.3	39.4^b^	45.3^b^	61.9^b^	44.9^b^	814.7^b^	325.8^b^
Variety (V)	513.5^b^	113.3^b^	631.0^b^	526.4^b^	128.3^b^	117.5^b^	365.9^b^	156.2^b^
Nitrogen(N)	3.5^a^	1.3	1.5	9.5^b^	24.4^b^	18.2^b^	24.3^b^	7.9^b^
Y × V	0.4	0.8	2.5	0.1	14.9^b^	0.1	18.8^b^	19.9^b^
Y × N	2.3	0.6	3.5^a^	2.0	2.0	2.9	9.7^b^	0.1
V × N	0.8	1.7	0.6	2.1	6.1^b^	5.2^a^	1.3	1.7
Y × V × N	0.2	1.2	0.2	0.1	0.1	1.7	0.2	1.2

Different lowercase letters represent a significant difference at the 0.05 level

*Abbreviations*: *CD* culm diameter, *CWT* culm wall thickness, *GCH* gravity centre height, *a*_*1*_ *and a*_*2*_ the outer and inner diameters of the minor axis in an oval cross-section, respectively, *b*_*1*_ *and b*_*2*_ the outer and inner diameters of the major axis in an oval cross-section, respectively, *PH* plant height

^a, b^ Means significant at the 0.05 and 0.01 probability levels, respectively

### Culm Anatomical Traits

Compared with W3668, the NLVB, NSVB, NAC, ALVB, ASVB and ACC of Wuyunjing23 were 10.2, 15.5, 9.4, 43.3, 71.1 and 73.5 % higher (Table 2). For both cultivars, an increase in top-dressing N application rates caused little variation in the NLVB, NSVB and NAC, but the ALVB and ASVB decreased by 23.4, 24.1 and 9.3, 24.0 % in the Wuyunjing23 and W3668 cultivars, respectively. Additionally, the interaction between the cultivar and N had significant effects on the ALVB (*F* = 5.7, *P* < 0.05) and ASVB (*F* = 13.0, *P* < 0.01). Higher N increased the AAC by 6.7 and 9.0 % in Wuyunjing23 and W3668 with no significant differences, respectively.

**Table 2:** Vascular bundle and air chambers of the N_4_ internode culm in two japonica rice cultivars under different nitrogen rates

Treatments	NLVB	ALVB (μm^2^)	NSVB	ASVB (μm^2^)	NAC	AAC (μm^2^)
Wuyunjing23						
LN	32.2a	21032.0a	32.8a	5594.5a	29.0a	21099.7a
MN	32.6a	19726.4a	31.3b	4706.5b	29.0a	21215.2a
HN	32.0a	16109.8b	32.5a	4245.1b	29.5a	22507.0a
W3668						
LN	29.6a	14002.8a	27.8a	3213.6a	27.2a	11850.3a
MN	29.3a	12986.7a	28.6a	2846.5a	26.2a	12072.6a
HN	28.8a	12706.5a	27.2a	2440.6b	26.8a	12915.9a
Analysis of variance						
Variety (V)	171.5^b^	139.6^b^	160.1^b^	598.8^b^	14.7^b^	64.1^b^
Nitrogen (N)	2.5	14.0^b^	0.7	32.0^b^	0.3	2.1
V × N	1.2	5.7^a^	6.0^a^	13.0^b^	0.2	0.6

Different lowercase letters represent a significant difference at the 0.05 level

*Abbreviations*: *NLVB* number of large vascular bundles, *ALVB* area of large vascular bundles, *NSVB*, number of small vascular bundles, *ASVB* area of small vascular bundles, *NAC* number of air chambers, *AAC* area of air chambers

^a, b^ Means significant at the 0.05 and 0.01 probability levels, respectively

**Fig. 3:**
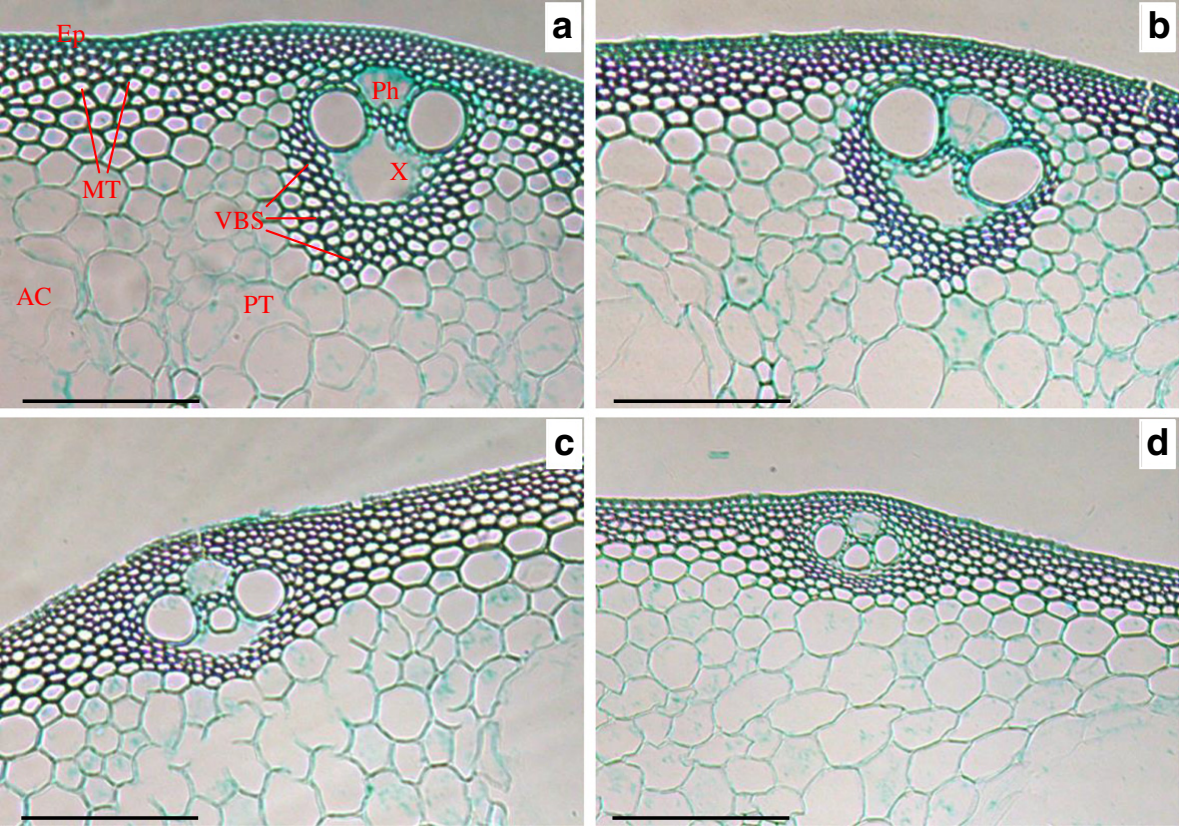
Anatomical characteristics of the N_4_ internode culm in two japonica rice cultivars under different nitrogen rates. **a** and **b** transverse sections at the second internode of the Wuyunjing23 cultivar under LN and HN conditions, respectively; **c** and **d** a transverse section at the second internode of the W3668 cultivar under LN and HN conditions, respectively. AC, air chamber; VBS, vascular bundles sheath; Ep, epidermis; MT, mechanical tissue; PT, parenchyma tissue X, xylem; Ph, phloem. Bar = 100 μm

**Fig. 4:**
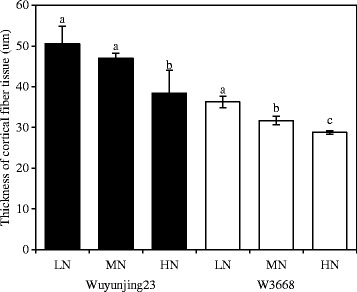
The thickness of the mechanical tissue of the N_4_ internodes culm in two japonica rice cultivars under different nitrogen rates. Different lowercase letters represent significant differences (*P* < 0.05) relative to the LN treatments for Wuyunjing23 and W3668, respectively

In the current study, the mechanical tissue and vascular bundle sheaths of the Wuyunjing23 cultivar were well-developed, whereas many hollow sclerenchyma cells were observed in the W3668 cultivar (Fig. 3). Top-dressing N rapidly altered the minute structures in the two japonica rice cultivars. For instance, with increasing top-dressing N application rates, the thickness of the mechanical tissue was remarkably reduced by 24.0 and 20.7 % in the Wuyunjing23 and W3668 cultivars, respectively (Fig. 4). Meanwhile, the layer of vascular bundle sheath cells in the Wuyunjing23 cultivar rapidly decreased under higher N rates (Fig. 3b). In the W3668 cultivar, the xylem and phloem were poorly developed, and the layer of mechanical tissue cells also decreased (Fig. 3d).

**Fig. 5:**
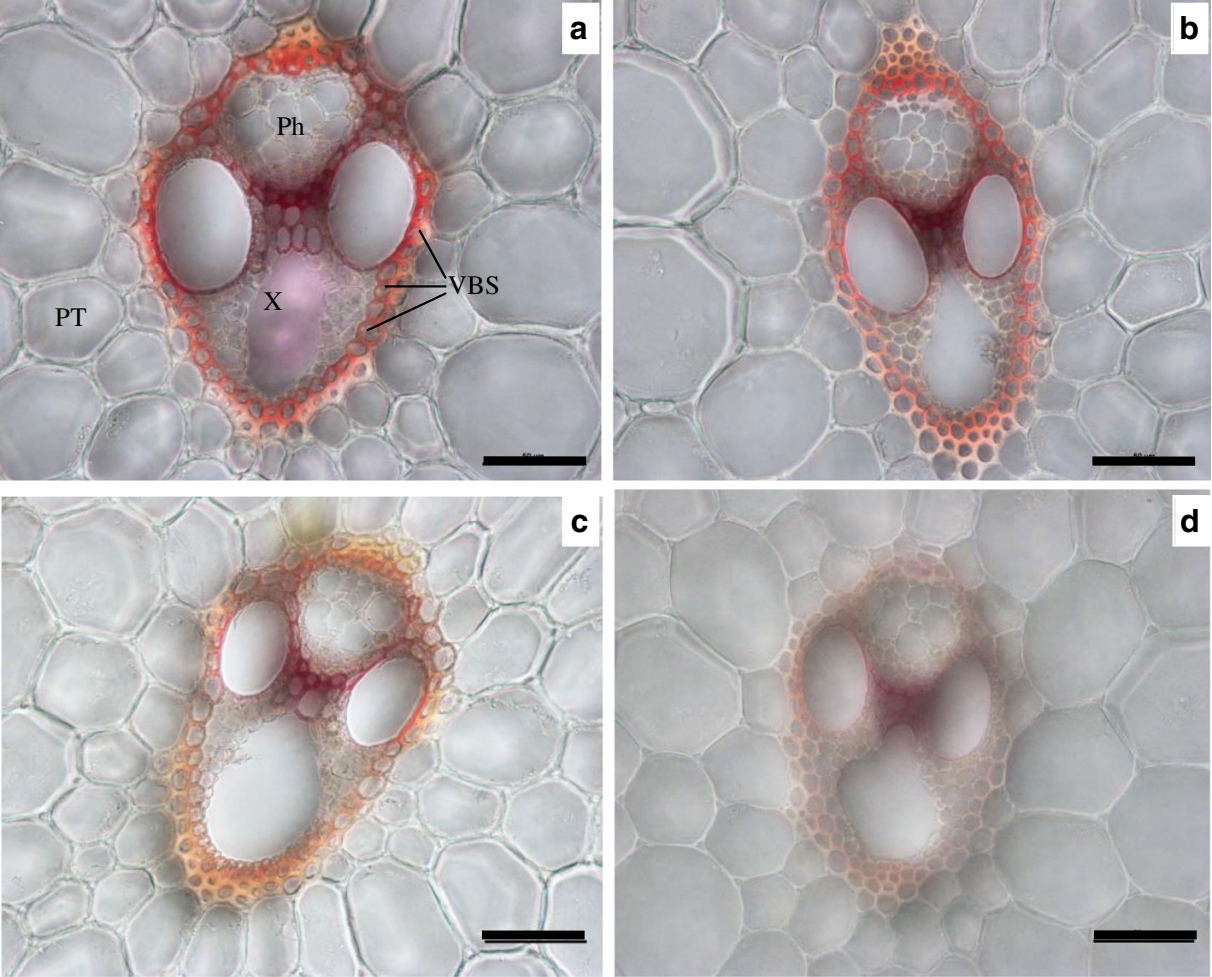
Large vascular bundle lignifications of the N_4_ internodes culm in two japonica rice cultivars under different nitrogen rates. **a** and **b** a transverse section at the second internode of the Wuyunjing23 cultivar under LN and HN conditions, respectively; **c** and **d** a transverse section at the second internode of the W3668 cultivar under LN and HN conditions, respectively Ph, Phloem; VBS, Vascular Bundle Sheath; X, Xylem; PT, Parenchyma Tissue, bar = 50 μm

The remarkable difference of large vascular bundles among different treatments was observed by using the phloroglucinol-HCl method, as shown in Fig. 5. With higher top-dressing N application rates, the large vascular bundle structure was altered. For instance, the ALVB of the Wuyunjing23 cultivar decreased rapidly, but lignifications of the vascular bundle sheath (VBS) decreased slightly under higher N rates (Fig. 5b). As for the W3668 cultivar, the xylem and phloem were poorly developed, and the VBS exhibited a collapsed structure due to lower lignin deposition in these cells (Fig. 5d).

### Correlation Analysis

In general, the lodging index was strongly and negatively correlated with breaking strength, with a correlation coefficient (r) of −0.987 (*P* < 0.01) for Wuyunjing23 and −0.938 (*P* < 0.01) for W3668, respectively (Table 3). Among the relationship between the breaking strength and anatomical traits, the breaking strength was closely and positively correlated with ALVB, ASVB and the thickness of mechanical tissue, with an r of 0.819 (*P* < 0.01), 0.820 (*P* < 0.01)  and 0.770 (*P* < 0.05) in Wuyunjing23 and an r of 0.707 (*P* < 0.05), 0.535 and 0.936 (*P* < 0.01) in W3668, respectively. Meanwhile, the breaking strength was slightly correlated with culm diameter and culm wall thickness but always negatively correlated with a_2_ and b_2_ in the two cultivars, with an r of −0.512, −0.816 (*P* < 0.01) in Wuyunjing23 and with an r of −0.599, −0.647 (*P* < 0.05) in W3668, respectively. In addition, the breaking strength was negatively correlated with ACC in both cultivars, with an r of −0.351, −0.679 (*P* < 0.05) in Wuyunjing23 and W3668, respectively.

**Table 3:** Correlation analysis between the physical parameters and the morphological and anatomical characteristics in japonica rice

Index	Wuyunjing23		W3668	
	LI	M	LI	M
Breaking strength (g.cm)	−0.987^b^	−	−0.938^b^	−
Plant height (cm)	0.578	−0.576	0.153	−0.071
Gravity centre height (cm)	0.588	−0.495	0.623	−0.429
Culm diameter (mm)	0.455	−0.440	0.725^a^	−0.586
Culm wall thickness (mm)	0.253	−0.164	−0.541	0.557
b_1_ (mm)	0.355	−0.311	0.800^b^	−0.671^a^
a_1_ (mm)	0.432	−0.405	0.934^b^	−0.798^b^
b_2_ (mm)	0.834^b^	−0.816^b^	0.795^a^	−0.647^a^
a_2_ (mm)	0.522	−0.512	0.484	−0.599
No. of large vascular bundles	0.155	−0.173	−0.486	0.322
Area of large vascular bundles (μm^2^)	−0.803^b^	0.819^b^	−0.666	0.707^a^
No. of small vascular bundles	−0.322	0.319	−0.12	−0.052
Area of small vascular bundles (μm^2^)	−0.870^b^	0.820^b^	−0.644	0.535
No. of air chambers	0.064	−0.100	0.074	−0.086
Area of air chambers (μm^2^)	0.401	−0.351	0.668^a^	−0.679^a^
Thickness of mechanical tissue (μm)	−0.759^a^	0.770^a^	−0.937^b^	0.936^b^

*Abbreviations*: *LI* lodging index, *M* breaking strength, *a*_*1*_ *and a*_*2*_ the outer and inner diameters of the minor axis in an oval cross-section, respectively, *b*_*1*_ *and b*_*2*_ the outer and inner diameters of the major axis in an oval cross-section, respectively

^a, b^ Means significant at the 0.05 and 0.01 probability levels, respectively

## Discussion

High N application rates, an important environmental factor, increases grain yield but decreases lodging resistance in rice plants by weakening the physical strength of the lower internodes (Wu et al., 2012; Zhang et al., 2015). Zhang WJ et al. (2014) indicated that N application rates decreased the breaking strength of culms by reducing bending stress in japonica super rice and decreasing section modulus in indica super rice. In wheat, Wei et al. (2008) suggested that the reduction of basal N application rates reduced the length of the lower internodes but increased the culm diameter, the culm wall thickness and the breaking strength, reducing the lodging index, which was consistent with a previous report by Lu et al. (2014). Our data showed that the culm wall thickness varied little, and the culm diameter increased rapidly in both cultivars of japonica rice with an increase in top-dressing N application rates (Table 1). However, the breaking strength was significantly reduced, which resulted in a higher lodging index (Fig. 1). These results are not consistent with previous reports (Ma et al., 2004; Kashiwagi et al., 2008; Yang et al., 2009). It is reasonable that the morphological traits of the basal stem were influenced by solar radiation reaching to the stem base, which largely depends on the leaf area index (LAI) and maximum tiller number at the panicle initiation stage (Li 1979; Ling et al., 1994). In this study, the maximum tiller number and LAI at the panicle initiation stage were almost identical under different top-dressing N application rates (data not shown); thus, the culm diameter might increase under higher N application rates at the panicle initiation stage, which is consistent with the reports by Li et al. (2013) and Wu et al. (2015).

Wang et al. (1998) reported that in wheat, the size of the medullary cavity, which is primarily affected by the inner diameter of the culm, was negatively correlated with lodging resistance. Our results show that top-dressing N significantly increased a_2_ and b_2_ in the culms of the two cultivars (Table 1). Moreover, correlation analysis indicated that a_2_ and b_2_ were positively correlated with the lodging index but negatively correlated with the breaking strength in both cultivars (Table 3). These results suggest that a smaller inner culm diameter might be helpful in enhancing the mechanical strength and would be responsible for lodging resistance in japonica rice.

Compared with 2013, plant height was significantly increased in 2014, which resulted in higher lodging risks in rice plants (Fig. 1). Previously, there were close relations between plant height and internode length (Sun 1987). In this study, the length of the lower internodes in 2014 was higher than in 2013, but the difference in the upper internodes was quite low; thus, the plant height and the lodging index were increased (Fig. 2). It was reasonable that higher solar radiation during the stem morphology formation stage (from panicle initiation to heading stage) in 2013 (21.5 MJ m^−2^ day^−1^) was significantly stronger than in 2014 (10.7 MJ m^−2^ day^−1^), which restrained the vertical elongation of plants and promoted lateral growth (Yang et al., 2009). These results were consistent with a previous report by Zhang J et al. (2014).

The variations of anatomical traits under different environments (such as nutrients, oxygen and light) enable plants to adapt to these conditions (Sultan, 2000; Santamaria, 2002). Nutrient availability, particularly nitrogen, has been widely suggested to affect plant growth by anatomical responses (Garnier et al., 1999; Grassein et al., 2010). Investigation of wheat, barley and oats showed that reducing nitrogen fertilizer rates induced the production of denser tissues, higher structural carbohydrates and lignin content in the lower stem (Mulder 1954). In addition, in cereal plants, vascular bundle tissues consisting of phloem, xylem and the vascular bundle sheaths are critical for the transport of water, minerals, nutrients and for mechanical support (Li et al., 2009). In this study, low top-dressing N increased the thickness of mechanical tissue and induced well-developed vascular bundle tissue in culms, which enhanced the stiffness and mechanical strength of the stem. By contrast, under high top-dressing N, the sclerenchyma cell layers under the epidermal layer and areas of vascular bundles were reduced (Fig. 3). Correlation analysis showed that the thickness of the mechanical tissue, the ALVB and the ASVB were significantly and negatively correlated with the lodging index but were significantly and positively correlated with the breaking strength (Table 3). These results suggest that a thicker mechanical tissue, ALVB and ASVB improves lodging resistance in japonica rice.

The higher the lodging index, the more susceptible the lodging response to N was. For the Wuyunjing23 cultivar, the response of the lodging rate in the field and the lodging index to top-dressing N tended to level off. With increasing top-dressing N application rates, the gravity centre height increased slightly but not significantly; however, the plant height increased higher (Table 1). Thus, to a certain extent, the lodging index of rice plants increased (Li et al., 2013; Zhang et al., 2015). Additionally, compared with LN treatments, the lignified cell walls in the mechanical tissue and vascular bundle sheath regions, which were closely related with stem stiffness, differed little (Figs. 3 and 5). However, the thickness of the mechanical tissue, the ALVB and the ASVB decreased significantly under HN conditions (Fig. 4, Table 2), resulting in a reduction in stem strength. These results were consistent with previous reports by Zhang et al. (2009). In addition, the culm inner diameter (a_2_, b_2_) increased with higher top-dressing N application rates, which were responsible for larger ratio of medullary cavity and weaker stem strength (Chen et al., 2008).

Under higher top-dressing N application conditions, the W3668 cultivar's mechanical tissue and vascular bundle sheath exhibited a remarkably loose structure (Figs. 3 and 5), and the ALVB and ASVB decreased (Table 2), suggesting a cell wall modification (either cellulosic or lignified) and a stem with a lower mechanical strength. These results can be explained as follows. Firstly, in our previous study, a large amount of non-structural carbohydrate (NSC) content was accumulated in the lower stem of the W3668 cultivar, but the structural carbohydrate (cellulose, lignin content) was quite low (Zhang et al., 2015). Secondly, the poor lignin accumulation in the basal culm and the weak stem strength under high N application were the results of lower related enzymes activities, such as phenylalanine ammonia-lyase (PAL), tyrosine-ammonia-lyase (TAL) and cinnamyl alcohol dehydrogenase (CAD) (Wang et al., 2012; Lu et al., 2014). Additionally, the AAC of the W3668 cultivar was increased by 9.0 % with a higher top-dressing N rate (Table 2), and it was negatively and significantly correlated with the breaking strength (Table 3). This result can be explained by the fact that high N levels are usually accompanied by low water oxygenation in a swamp environment (Camargo and Alonso 2006). The good development of aerenchyma in plants may represent an adaptation to the hypoxic conditions induced by N enrichment as it improves gas diffusion through the plant, thus increasing the respiration rate (Hussner et al., 2009; Ryser et al., 2011). However, the aerenchyma is a mechanically weak tissue providing little stiffness to the plant (Striker et al., 2007).

## Conclusions

The shorter length of the lower internodes, smaller inner culm diameter and higher thickness of mechanical tissue in japonica rice improved stem strength and reduced lodging risk. The lodging resistance-related morphological and anatomical trait response to top-dressing N differed by japonica rice genotype. With higher top-dressing N, the plant height and inner culm diameter of the Wuyunjing23 cultivar increased, but the ALVB and ASVB decreased. The mechanical tissue and vascular bundle of the W3668 cultivar exhibited a loose structure due to deficient lignified cell walls, a decreased ALVB and ASVB, and an increased AAC, which resulted in a lower stem strength and a higher lodging index.
