# Erratum to: ‘Lodging Resistance of Japonica Rice (*Oryza Sativa* L.): Morphological and Anatomical Traits Due to Top-Dressing Nitrogen Application Rates’

**DOI:** 10.1186/s12284-016-0110-9

**Published:** 2016-07-27

**Authors:** Wujun Zhang, Longmei Wu, Xiaoran Wu, Yanfeng Ding, Ganghua Li, Jingyong Li, Fei Weng, Zhenghui Liu, She Tang, Chengqiang Ding, Shaohua Wang

**Affiliations:** 1 Jiangsu Collaborative Innovation Center for Modern Crop Production/National Engineering and Technology Center for Information Agriculture/ Key Laboratory of Crop Physiology and Ecology in Southern China, Nanjing Agricultural University, Nanjing, 210095 China; 2 Chongqing Academy of Agricultural Sciences/Chongqing Ratooning Rice Research Center, Chongqing, 402160 China

## Erratum

Unfortunately, the original version of this article (Zhang et al. 2016) contained an error. An author’s name was included incorrectly in this article. The author’s name was recorded as Longwei Wu and it should have been recorded as Longmei Wu. There will also be an update to correct this error.
